# Deficiency of Prdm13, a dorsomedial hypothalamus-enriched gene, mimics age-associated changes in sleep quality and adiposity

**DOI:** 10.1111/acel.12299

**Published:** 2014-12-25

**Authors:** Akiko Satoh, Cynthia S Brace, Nick Rensing, Shin-ichiro Imai

**Affiliations:** 1 Department of Developmental Biology, Washington University School of MedicineSt. Louis, MO, 63110, USA; 2 Department of Neurology, Washington University School of MedicineSt. Louis, MO, 63110, USA

**Keywords:** aging, age-associated pathophysiology, Dorsomedial hypothalamus, DMH-enriched gene, Nkx2-1, NREM delta power, Prdm13

## Abstract

The dorsomedial hypothalamus (DMH) controls a number of essential physiological responses. We have demonstrated that the DMH plays an important role in the regulation of mammalian aging and longevity. To further dissect the molecular basis of the DMH function, we conducted microarray-based gene expression profiling with total RNA from laser-microdissected hypothalamic nuclei and tried to find the genes highly and selectively expressed in the DMH. We found *neuropeptide VF precursor (Npvf)*,*PR domain containing 13 (Prdm13)*, and *SK1 family transcriptional corepressor (Skor1)* as DMH-enriched genes. Particularly, Prdm13, a member of the Prdm family of transcription regulators, was specifically expressed in the compact region of the DMH (DMC), where Nk2 homeobox 1 (Nkx2-1) is predominantly expressed. The expression of *Prdm13* in the hypothalamus increased under diet restriction, whereas it decreased during aging. *Prdm13* expression also showed diurnal oscillation and was significantly upregulated in the DMH of long-lived BRASTO mice. The transcriptional activity of the *Prdm13* promoter was upregulated by Nkx2-1, and knockdown of *Nkx2-1* suppressed *Prdm13* expression in primary hypothalamic neurons. Interestingly, DMH-specific *Prdm13*-knockdown mice showed significantly reduced wake time during the dark period and decreased sleep quality, which was defined by the quantity of electroencephalogram delta activity during NREM sleep. DMH-specific *Prdm13*-knockdown mice also exhibited progressive increases in body weight and adiposity. Our findings indicate that Prdm13/Nkx2-1-mediated signaling in the DMC declines with advanced age, leading to decreased sleep quality and increased adiposity, which mimic age-associated pathophysiology, and provides a potential link to DMH-mediated aging and longevity control in mammals.

## Introduction

The hypothalamus is structurally divided into several functionally distinct areas called nuclei, such as the arcuate, ventromedial, dorsomedial, lateral, paraventricular, and suprachiasmatic nuclei of the hypothalamus (Arc, VMH, DMH, LH, PVN, and SCN, respectively). Each hypothalamic nucleus plays a crucial role in the regulation of various physiological responses, including feeding behavior, metabolism, endocrine regulation, physiological rhythm, emotion, and aging (Satoh & Imai, 2014). Recent findings demonstrate that the hypothalamus functions as a high-order ‘control center of aging,’ counteracting age-associated functional changes and thereby promoting longevity in mammals (Satoh *et al*., 2013; Zhang *et al*., 2013). Zhang *et al*. demonstrated that NF-κB signaling in the mediobasal hypothalamus (MBH) is significantly enhanced with advanced age, and suppressing NF-κB signaling in the MBH retards aging and extends lifespan in mice (Zhang *et al*., 2013). We also demonstrated that the mammalian NAD^+^-dependent protein deacetylase Sirt1 in the hypothalamus, particularly the DMH and LH, is critical to counteract age-associated declines in skeletal muscle mitochondrial function, physical activity, body temperature, oxygen consumption, and sleep quality, and promote longevity in mice (Satoh *et al*., 2013). Remarkably, increasing Sirt1 dosage only in the DMH is sufficient to regain physical activity in aged mice to a similar level as in young mice (Satoh *et al*., 2013). These results clearly demonstrate the importance of the hypothalamus, more specifically DMH function, in controlling mammalian aging.

Aging has a significant impact on DMH function. The DMH has been known to regulate body temperature (Zaretskaia *et al*., 2003; Morrison *et al*., 2008; Enriori *et al*., 2011), food-anticipating activity (Gooley *et al*., 2006; Acosta-Galvan *et al*., 2011), autonomic stress responses (Ulrich-Lai & Herman, 2009), food intake (Yang *et al*., 2009), and reproduction (Kirby *et al*., 2009; Soga *et al*., 2014). Studies illustrate several examples of the decline in DMH function with age. The capacity of thermoregulation declines with age (Reynolds *et al*., 1985). In humans, it has been reported that mean temperature declines with age, after controlling for sex, body mass index, and white blood cell count (Waalen & Buxbaum, 2011). Food-anticipating activity in response to timed-restricted feeding also declines in old rats (Shibata *et al*., 1994). The peripheral hormone ghrelin activates the DMH neurons, causing the enhancement of food intake, and this enhancement by ghrelin declines in old mice (Akimoto & Miyasaka, 2010). Finally, it has been reported that the ability of the reproductive system declines with advanced age, partly due to the reduction of DMH function (Soga *et al*., 2014). Given those findings, it is clear that DMH function declines over age. Thus, it is important to know the mechanisms by which the DMH controls these functions to treat and/or prevent age-associated pathophysiology.

To dissect the molecular basis of DMH function, we decided to search for the genes highly and selectively expressed in the DMH. Transcriptome studies have so far shown that particular subsets of neuronal genes exhibit clear spatial expression patterns across the adult mouse brain, suggesting that these spatial expression patterns are related to the unique physiological functions of distinct brain regions (Wang *et al*., 2010; Ko *et al*., 2013). Whereas other hypothalamic nuclei such as the Arc, VMH, LH, and PVN have been well characterized for their genetic and chemical identities (Elmquist *et al*., 1999; Leak & Moore, 2001; Gottsch *et al*., 2004; Segal *et al*., 2005), efforts to characterize the DMH have been very limited. Lee *et al*. (2012) reported a comprehensive gene profile for the ventral subdivision of the DMH (DMV) where the leptin receptor is exclusively expressed (Zhang *et al*., 2011). Although the DMH-enriched genes identified in this study are only moderately changed by dietary manipulations (Lee *et al*., 2012), subsequent studies have proven the importance of these genes in response to leptin (Bechtold *et al*., 2012). These findings indicate the potential for exploring the DMH-enriched genes to further dissect the physiological role of the DMH in neurobehavioral and metabolic regulation.

In this study, we identified several genes that display DMH-enriched expression patterns by performing gene expression profiling with RNA samples collected from laser-microdissected hypothalamic nuclei. We examined the distribution of these candidate genes in the subcompartments of the DMH and evaluated changes in gene expression under diet restriction (DR), through the diurnal cycle, and with age. Among those genes, we identified Prdm13, a member of the Prdm family (Fog *et al*., 2012; Hohenauer & Moore, 2012), as a highly DMH-enriched gene. The expression of *Prdm13* increased under DR, whereas its expression decreased with advanced age. Moreover, the expression level of *Prdm13* in the DMH of long-lived brain-specific *Sirt1*-overexpressing transgenic (BRASTO) mice is significantly higher than the level in wild-type control mice. Interestingly, we found that mice with DMH-specific knockdown of *Prdm13* displayed a robust decline in sleep quality and a progressive increase in body weight. These findings provide a novel possibility that a specific subset of neurons, characterized by the expression of DMH-enriched genes, regulate sleep homeostasis and metabolism and that the functional deterioration of those specific DMH neurons could contribute to age-associated pathophysiology in mammals.

## Results

### Identification of genes enriched in the Arc, VMH, DMH, and LH

**Figure 1:**
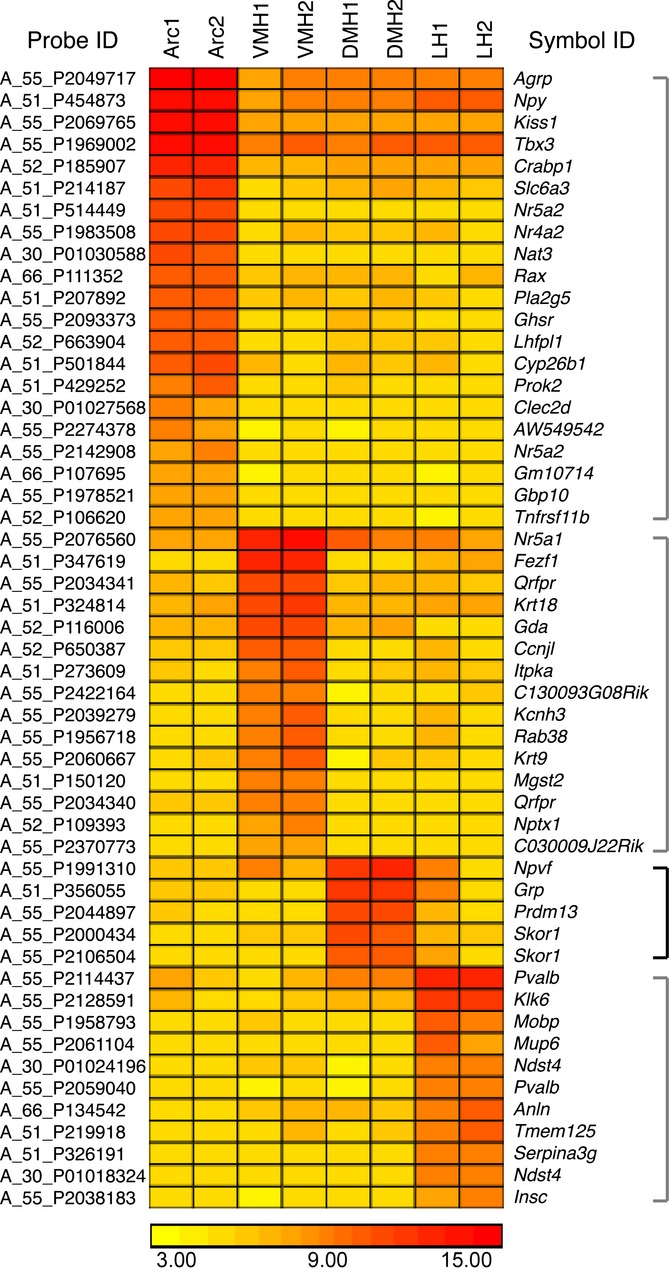
Heat map representation of genes enriched in the Arc, VMH, DMH, and LH. Genes expressed greater than or equal to 10-fold in each hypothalamic nucleus compared to other hypothalamic nuclei by calculating ratios of signal intensities between each hypothalamic nucleus are shown in the heat map. Microarray analysis was conducted using two individual RNA samples from the Arc (Arc1 and Arc2), VMH (VMH1 and VMH2), DMH (DMH1 and DMH2), and LH (LH1 and LH2). Expression levels are indicated by the colored log2 scale of signal intensities shown at the bottom of the figure. Probe ID from the Agilent Mouse_v1 8x60K array (left) and corresponding symbol ID (right) are shown for each gene.

To identify genes selectively expressed in the DMH, we collected RNA samples from four hypothalamic nuclei, namely the Arc, VMH, DMH, and LH, using laser microdissection (Fig. S1) and conducted microarray analysis to compare their gene expression profiles. We determined genes selectively expressed greater than or equal to 10-fold in the Arc, VMH, DMH, and LH, respectively, compared to the other hypothalamic nuclei by calculating ratios of the signal intensities between each hypothalamic nucleus. Using this criterion, 21, 15, four, and eleven genes were found as genes enriched in the Arc, VMH, DMH, and LH, respectively (Fig.1 and Table S1). We identified *neuropeptide VF precursor (Npvf)* (NM_021892), *gastrin-releasing peptide* (*Grp)* (NM_175012), *PR domain containing 13 (Prdm13)* (NM_001080771), and *SK1 family transcriptional corepressor (Skor1)* (NM_172446) as the DMH-enriched genes (Table1). Our analysis also confirmed that *agouti-related protein* (*Agrp*) (NM_007427), *neuropeptide Y* (*Npy*) (NM_023456), and *kisspeptin 1* (*Kiss1*) (NM_178260) are exclusively expressed in the Arc, and *nuclear receptor subfamily 5, group A, member 1* (*Nr5a1*) (NM_139051) is exclusively expressed in the VMH (Table S1). These genes are well-known selective markers of the Arc and VMH (Elmquist *et al*., 1999; Gottsch *et al*., 2004; Segal *et al*., 2005), providing further validation of our results. *Parvalbumin* (*Pvalb*) (NM_013645) is among the top genes that show much higher expression levels in the LH compared to the other hypothalamic nuclei (Table S1), which was also confirmed by the Allen Brain Atlas (http://mouse.brain-map.org/), although *Pvalb* is also highly expressed in other brain regions such as the cerebellum and cortex.

**Table 1:** Genes highly and selectively expressed in the DMH

Probe ID	Acquisition number	Arc1	Arc2	VMH1	VMH2	DMH1	DMH2	LH1	LH2	
A_55_P1991310	NM_021892	75	82	369	92	4610	5816	427	25	*Npvf*
A_51_P356055	NM_175012	75	67	27	27	3242	5527	395	28	*Grp*
A_55_P2044897	NM_001080771	68	26	27	25	1942	2026	147	24	*Prdm13*
A_55_P2000434	NM_172446	27	25	60	87	1655	1425	170	56	*Skor1*
A_55_P2106504	NM_172446	25	23	26	24	1402	1363	193	25	*Skor1*

Numbers indicate signal intensities from microarray analysis.

### Expression patterns of *Npvf*,*Prdm13*, and *Skor1* in the brain, in the hypothalamus, and within the DMH

**Figure 2:**
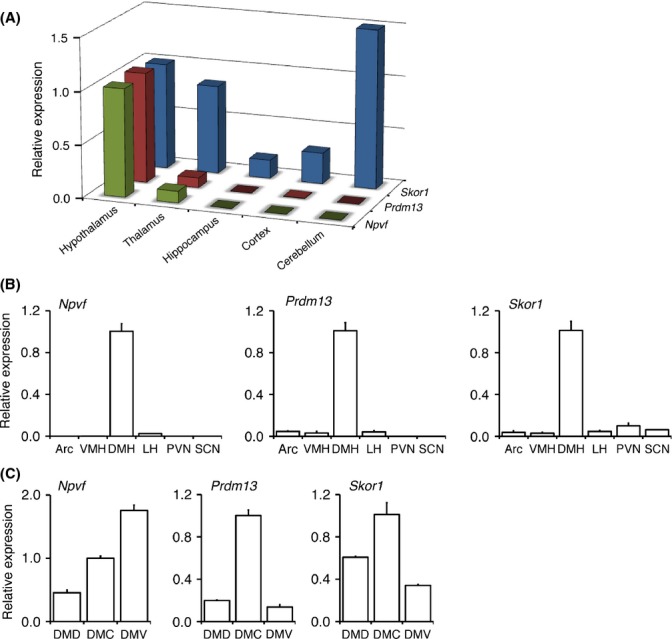
Distribution of *Npvf*,*Prdm13*, and *Skor1* in the brain and specifically within the hypothalamus. (A, B, and C) RNA expression levels of *Npvf*,*Prdm13*, and *Skor1* were examined in various brain regions including the hypothalamus, thalamus, hippocampus, cortex, and cerebellum (A), in the hypothalamic nuclei including the Arc, VMH, DMH, LH, PVN, and SCN (B) and in the DMH subdivisions including the DMD, DMC, and DMV (C). Expression levels were normalized to those of the hypothalamus (A), DMH (B), or DMC (C). Results are shown as mean ± S.E. (*n* = 2–3 mice for each group).

We next determined the anatomical distribution of these DMH-enriched genes in the brain. Exploring DMH-enriched genes will have a potential to further address DMH function because the analysis of cell-type-specific gene expression patterns could elucidate brain function (Wang *et al*., 2010; Ko *et al*., 2013). We excluded Grp from the DMH-enriched genes we identified because it is also highly expressed in other peripheral tissues based on data available from the gene annotation portal BioGPS (http://biogps.org/#goto=welcome). We examined whether the expression of *Npvf*,*Prdm13*, and *Skor1* was restricted to the hypothalamus. qRT-PCR was conducted with total RNAs from the hypothalamus, thalamus, hippocampus, cortex, and cerebellum. We found that *Npvf* and *Prdm13* were selectively localized in the hypothalamus, whereas *Skor1* was expressed in the hypothalamus, thalamus, hippocampus, cortex, and most extensively in the cerebellum (Fig.2A). We also examined the expression profiles of these three genes through the hypothalamic nuclei including the Arc, VMH, DMH, LH, PVN, and SCN. Consistent with the data from our microarray analysis, *Npvf*,*Prdm13,* and *Skor1* were exclusively expressed in the DMH (Fig.2B). The DMH is further divided into three subdivisions: dorsal, compact, and ventral DMH (DMD, DMC, and DMV, respectively) (Fig. S1A). The highest expression of *Npvf* was observed in the DMV compared to the other two DMH subdivisions, whereas the expression levels of *Prdm13* and *Skor1* were highest in the DMC (Fig.2C).

### Expression changes in *Npvf*,*Prdm13*, and *Skor1* in the hypothalamus under DR, through the 24-h diurnal cycle, and with age

**Figure 3:**
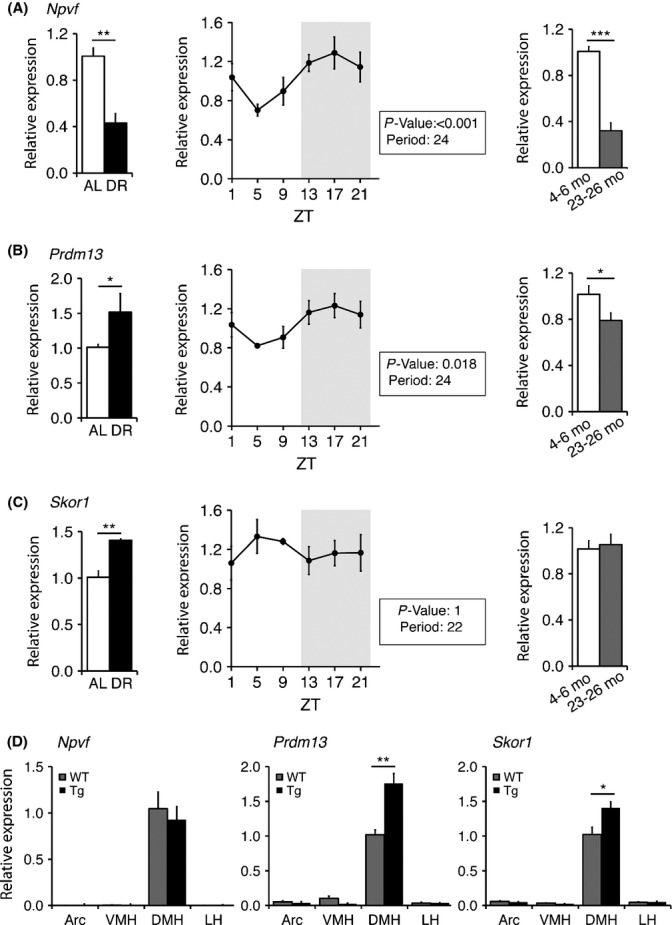
Alteration of *Npvf*,*Prdm13*, and *Skor1* expression levels under DR, through the 24-h diurnal cycle, during the aging process, and in aged BRASTO mice. RNA expression levels of *Npvf* (A), *Prdm13* (B), and *Skor1* (C) in the hypothalamus were examined under *ad libitum* (AL)-fed versus diet restriction (DR) conditions at *Zeitgeber time* (ZT) 3 (left), every 4 h at different ZT points (middle), and in young (4–6 months of age) versus old (23–26 months of age) mice at ZT15 (right). Expression levels were normalized to those of AL (left) or young (right) groups. (D) RNA expression levels of *Npvf*,*Prdm13*, and *Skor1* in the Arc, VMH, DMH, and LH of BRASTO mice (Tg) and wild-type mice (WT) at 20 months of age at ZT15. Results are shown as mean ± S.E. (*P < 0.05, **P < 0.01, ***P < 0.001, *n* = 4 mice each for AL and DR,*n* = 3–4 mice for each time point through 24 h, *n* = 7–8 mice for each group young vs. old, *n* = 4 mice each genotype). Shaded area represents the dark period.

As our recent findings indicate the importance of the DMH in the regulation of central adaptive responses to DR, physiological rhythms, and aging and longevity (Satoh *et al*., 2010, 2013), it is likely that these DMH-enriched genes show interesting expression changes in these conditions. To address this possibility, we first analyzed expression levels of *Npvf*,*Prdm13*, and *Skor1* in the hypothalamus under DR, an experimentally proven dietary regimen that delays the aging process and extends lifespan in a wide variety of organisms (Guarente, 2013). The level of *Npvf* significantly decreased to about 40% under DR compared to that of *ad libitum* (AL)-fed controls (Fig.3A, left), whereas levels of *Prdm13* and *Skor1* significantly increased about 50% and 45%, respectively (Figs.3B and C, left). We next examined the diurnal expression patterns of *Npvf*,*Prdm13,* and *Skor1*. In the hypothalamus, both *Npvf* and *Prdm13* displayed statistically significant 24-h diurnal cycles, with higher expression during the dark period and lower expression during the light period (Figs.3A and B, middle). The robust oscillation was not observed in the expression of *Skor1* in the hypothalamus (Fig.3C, middle). Lastly, we compared the expression of *Npvf*,*Prdm13*, and *Skor1* in the hypothalamus between mice at 4–6 months and 23–26 months of age. As the expression of *Npvf* and *Prdm13* increases during the dark period, we collected hypothalamic samples from young and old mice during the dark period. The expression levels of *Npvf* in the hypothalamus significantly decreased in mice at 23–26 months of age, similar to its reduction under DR (Fig.3A, right). Intriguingly, the expression of *Prdm13* significantly decreased with age, whereas its expression was elevated under DR (Fig.3B, right). The level of *Skor1* was indistinguishable between young and old mice (Fig.3C, right). We also measured the expression levels of *Npvf*,*Prdm13*, and *Skor1* in hypothalamic nuclei (Arc, VMH, DMH, and LH) of aged BRASTO mice, which showed significant delay in aging and lifespan extension (Satoh *et al*., 2013). Interestingly, the levels of *Prdm13* and *Skor1* in the DMH of aged BRASTO mice were significantly higher than the levels in wild-type control mice, whereas the levels of *Npvf* in the DMH were indistinguishable between genotypes (Fig.3D). These results suggest a possibility that *Prdm13* and *Skor1* contribute to the BRASTO phenotypes. Given that the data from DR, 24-h diurnal cycle, aging, and aged BRASTO mice, *Prdm13* might be a key to counteract age-associated functional declines and to promote lifespan in mammals (Satoh *et al*., 2013).

### The Nk2 homeobox 1 transcription factor Nkx2-1 shows DMC-predominant expression in the DMH and upregulates *Prdm13* transcription

**Figure 4:**
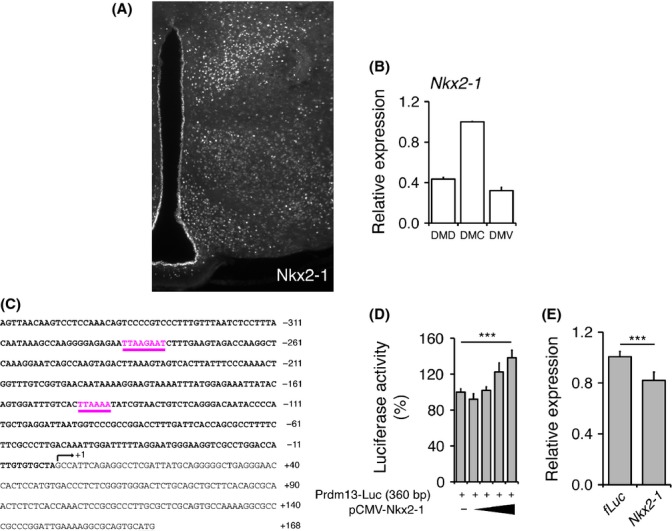
*Prdm13* as a downstream gene regulated by *Nkx2-1*. (A) Immunohistochemistry staining of Nkx2-1 in the hypothalamus. White dots represent staining with an anti-Nkx2-1 antibody. We confirmed that these Nkx2-1-positive cells are NeuN-positive neurons (Fig. S2). (B) RNA expression levels of *Nkx2-1* in the DMH subdivisions (DMD, DMC, and DMV). Expression levels were normalized to those of DMC. Results are shown as mean ± S.E. (*n* = 3 mice per subdivision). (C) Nucleotide sequence of the 360-bp upstream region of the mouse *Prdm13* gene. The transcription initiation site is defined as +1, and the start codon (ATG) is underlined. Predicted Nk2 family binding sites are shown and underlined in pink color. (D) Luciferase activities driven by the 360-bp *Prdm13* promoter fragment [*Prdm13*-Luc (360 bp)] in HEK293 cells cotransfected with Nkx2-1 (pCMV-Nkx2-1). Luciferase activities in cells cotransfected with a backbone vector were normalized to 100%. Results are shown as mean values ± S.E. (***P < 0.001 by one-way ANOVA with Tukey–Kramer *post hoc* test, *n* = 9). (E) RNA expression levels of *Prdm13* after knocking down *Nkx2-1* in primary hypothalamic neurons. Expression levels were normalized to those of *firefly Luciferase* (*fLuc*)-knockdown controls' cells. Results are shown as mean values ± S.E. (***P < 0.001 by Student's *t*-test, *n* = 7).

Interestingly, as well as *Prdm13*, Nkx2-1, a substrate and binding partner of Sirt1 in the DMH (Satoh *et al*., 2013), also displayed DMC-predominant expression. Immunohistochemistry using a specific antibody against Nkx2-1 revealed that Nkx2-1 is highly expressed in the DMC compared to other hypothalamic nuclei (Fig.4A). We also confirmed that Nkx2-1 is colocalized with NeuN in the DMC (Figs. S2A–D). qRT-PCR analysis clearly indicated that *Nkx2-1* is most highly expressed in the DMC among the DMH subdivisions (Fig.4B). Given this anatomical overlap between *Prdm13* and *Nkx2-1* and the diurnal oscillation of *Prdm13* expression, we hypothesized that *Prdm13* could be a transcriptional target of Nkx2-1, whose activity is regulated by Sirt1 in a diurnal manner (Satoh *et al*., 2013). We found two predicted Nk2 binding sites in the *Prdm13* promoter region (Fig.4C). To confirm whether the *Prdm13* promoter indeed responds to Nkx2-1, we generated a luciferase reporter construct with the 360-bp genomic fragment of the *Prdm13* promoter that contains these two Nk2 binding motif sequences. Increasing *Nkx2-1* expression significantly enhanced the transcriptional activity of this *Prdm13* promoter fragment in a dose-dependent manner in HEK293 cells (Fig.4D). Therefore, we next tested whether *Nkx2-1* is sufficient to promote *Prdm13* expression in primary hypothalamic neurons. Knocking down *Nkx2-1* about 85–90% (data not shown) significantly but moderately reduced *Prdm13* expression in the hypothalamic neurons (Fig.4E). These results suggest that *Prdm13* transcription is regulated by *Nkx2-1*, although other factors or regulatory mechanisms are involved in the transcriptional regulation of *Prdm13*.

### *Prdm13* in the DMH affects sleep quality and body weight

**Figure 5:**
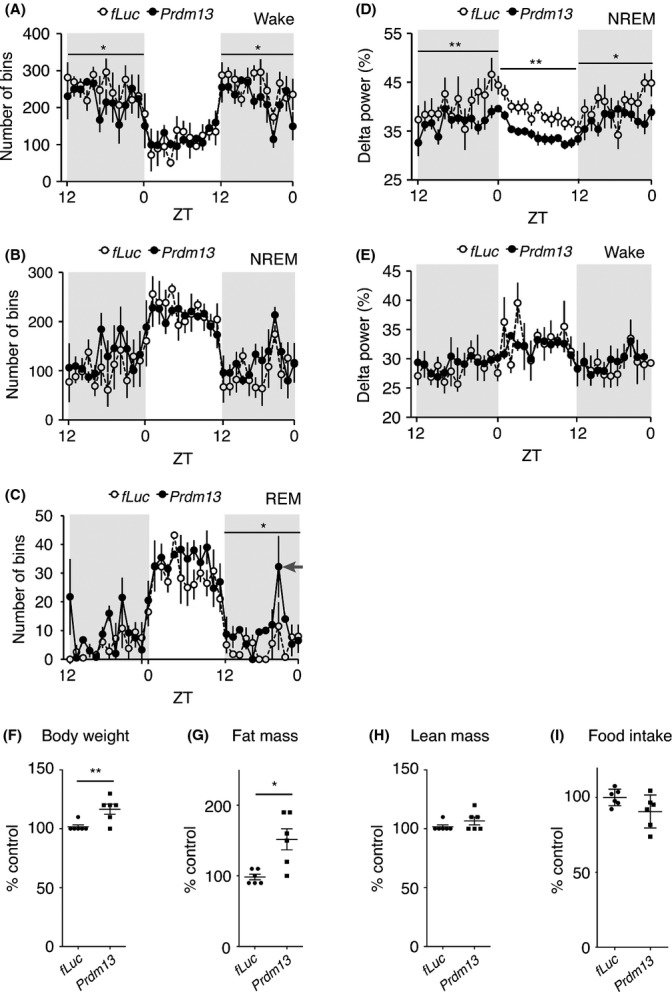
Sleep–wake patterns, NREM delta power, body weight, body composition, and daily food intake in DMH-specific *Prdm13*-knockdown mice. (A–C) The amount of wakefulness (wake) (A), NREM sleep (NREM) (B), and REM sleep (REM) (C) in DMH-specific *firefly Luciferase* (*fLuc*)-knockdown control mice and *Prdm13*-knockdown mice. Numbers of bins are shown as mean values ± S.E. (*P < 0.05 by Wilcoxon matched-pairs signed-rank test, *n* = 5–6 mice per group). Shaded areas represent the dark periods. A gray arrow in C indicates the ZT20 time point on Day 2 when an abnormal peak was observed in *Prdm13*-knockdown mice (see text). (D and E) Electroencephalogram delta power in NREM sleep (D) or in wake (E). Data are shown as mean values ± S.E. (*P < 0.05, **P < 0.01 by Wilcoxon matched-pairs signed-rank test, *n* = 5–6 mice per group). (F–H) Body weight (F), fat mass (G), lean mass (H), and daily food intake (I) for *Prdm13*-knockdown mice are normalized to the average values of *fLuc*-knockdown control mice within each cohort (3 cohorts). Fat mass and lean mass were measured by EchoMRI. Individual data are plotted, and mean values and S.E. are shown (*P < 0.05, **P < 0.01 by Student's *t*-test, *n* = 6 mice per group).

To further elucidate the physiological significance of *Prdm13* in the DMH, we generated DMH-specific *Prdm13*-knockdown mice by stereotactic injection of lentiviruses carrying *Prdm13* shRNA. We then conducted electroencephalogram (EEG) recording on these mice. DMH-specific *Prdm13*-knockdown mice and control shRNA-injected mice showed indistinguishable sleep/wake architecture (Fig.5A–C), except that *Prdm13-*knockdown mice displayed a significantly lower amount of wakefulness during the dark period compared to controls on both Day 1 and Day 2 (Fig.5A). This reduction of wakefulness in *Prdm13-*knockdown mice was not accompanied by an alteration in the amount of NREM sleep (Fig.5B). We also found that the amount of REM sleep was significantly higher in *Prdm13*-knockdown mice compared to controls during the dark period only on Day 2 (Fig.5C). It should be noted that this statistically significant difference on Day 2 was dependent on a single data point showing an abnormal peak at ZT20 (shown as arrow in Fig.5C). Additionally, this difference was not observed on Day 1. These results suggest that *Prdm13* in the DMH has a minimal effect on the normal diurnal oscillation of sleep. On the other hand, we found that the level of EEG delta power during NREM sleep, an indicator of the quality or depth of sleep, was significantly reduced in DMH-specific *Prdm13*-knockdown mice compared to control mice during both light and dark periods (Fig.5D), whereas these differences were not observed during the wake time (Fig.5E). This delta power defect caused by *Prdm13* knockdown is very similar to that caused by *Nkx2-1* knockdown (Satoh *et al*., 2013). Thus, these results indicate that *Prdm13* in the DMH, as well as its upstream regulator Nkx2-1 (Satoh *et al*., 2013), significantly affects the level of sleep depth or quality in mice.

Poor sleep quality contributes to excessive weight gain, and better sleep quality confers protection against obesity (Mavanji *et al*., 2010). Therefore, we measured body weight in DMH-specific *Prdm13*-knockdown and control mice 2.5 months after stereotactic surgery. Because actual measured values of body weight, fat mass, and lean mass in control and *Prdm13*-knockdown mice fluctuated between cohorts, we calculated the percent changes compared to control mice in each individual cohort. The relative values of body weight and fat mass in *Prdm13*-knockdown mice were significantly higher than those in control mice (Fig.5F and G), whereas relative values of lean mass did not differ between control and *Prdm13*-knockdown mice (Fig.5H). We also observed that the amounts of daily food intake were indistinguishable between these two groups, suggesting that the increases in body weight and fat mass in *Prdm13*-knockdown mice are not due to increased daily food intake. These results indicate that DMH-specific *Prdm13*-knockdown mice exhibit progressive gain of adipose tissue when their sleep quality is affected.

## Discussion

In our present study, we identified three DMH-enriched genes, *Npvf*,*Prdm13*, and *Skor1,* from microarray analysis using laser-microdissected hypothalamic nuclei. Intriguingly, *Prdm13* is enriched in the DMC, where Sirt1 and Nkx2-1 are highly colocalized (Satoh *et al*., 2013). Indeed, the transcriptional activity of the *Prdm13* promoter is upregulated by Nkx2-1, and the knockdown of *Nkx2-1* decreases *Prdm13* expression in primary hypothalamic neurons. Interestingly, the expression of *Prdm13* increases under DR, whereas its expression declines with age. In addition, the expression level of *Prdm13* in the DMH of aged BRASTO mice is significantly higher than the level in wild-type control mice. Furthermore, DMH-specific *Prdm13*-knockdown mice display reduced sleep quality and increased body weight, phenotypes very similar to old animals (Ancoli-Israel, 2009; Kuk *et al*., 2009). Given that Sirt1 and Nkx2-1 function together in the hypothalamus to counteract age-associated functional declines (Satoh *et al*., 2013), *Prdm13* might also play an important role as one of their key target genes in the DMH to mediate age-associated pathophysiology, particularly sleep quality and body weight.

Different from other hypothalamic nuclei, it has been challenging to identify selective marker genes for the DMH. Lee *et al*. previously reported *Gpr50*,*Pcsk5*,*Sulf1, Grp,* and *Ror*β as genes highly expressed in the DMV (Lee *et al*., 2012). Our microarray analysis did not identify those genes, except for *Grp*, which is highly expressed in other peripheral tissues. This is likely due to differences in sample collection between the two studies: DMV in their study versus DMH in our study. In fact, *Prdm13* and *Skor1* are highly expressed in the DMC, but not in the DMV. *Npvf* could have been detected in their study because *Npvf* is highly expressed in the DMV based on our results. Kasukawa *et al*. also reported *Npvf* as a potential candidate maker for the DMH, based on the BrainStars database that they created (http://brainstars.org/) (Kasukawa *et al*., 2011). Additionally, the localization of *Npvf* in the DMV can be confirmed in the Allen Brain Atlas (http://mouse.brain-map.org/). Taken together, *Npvf* and *Prdm13* are specifically expressed in the DMV and DMC, respectively, potentially providing good selective markers for the DMH.

The physiological role of Prdm13 during adulthood is still poorly understood. Prdm13 belongs to the Prdm family of zinc finger transcriptional regulators (Fog *et al*., 2012; Hohenauer & Moore, 2012). Recently, it has been reported that Prdm13 has histone methyltransferase activity (Hanotel *et al*., 2014). During development, Prdm13 is directly downstream of Ptf1a, a basic helix–loop–helix transcription activator and is required for the specification of GABAergic and glutamatergic neurons (Chang *et al*., 2013). Remarkably, we demonstrated that Prdm13 in the DMH is important to maintain sleep quality and body weight associated with adiposity. DMH-specific *Prdm13*-knockdown mice displayed decreased wakefulness during the dark period and robust decline in NREM delta power through the entire diurnal cycle compared to those in control mice. They also displayed progressively increased body weight with adiposity. Because sleep quality and metabolism are highly associated (Morselli *et al*., 2012), poor sleep quality by knockdown of *Prdm13* in the DMH might cause increased body weight secondarily. Alternatively, weight gain with adiposity might be regulated by the same set of DMH neurons but through an independent mechanism (see below). Sleep quality is regulated by circadian rhythmicity and sleep–wake homeostasis. Provided that NREM delta power is regulated independently of sleep duration (Davis *et al*., 2011), the observed abnormality of sleep in DMH-specific *Prdm13*-knockdown mice might be due to defects in sleep–wake homeostasis. The DMH has been suggested to be a relay point for signals going from the SCN to the locus coeruleus (Aston-Jones *et al*., 2001), the main site for synthesizing norepinephrine in the brain and facilitating arousal and sleep–wake homeostasis. Thus, it will be interesting to examine whether DMH-specific *Prdm13*-knockdown mice also display defects in sleep homeostasis.

**Figure 6:**
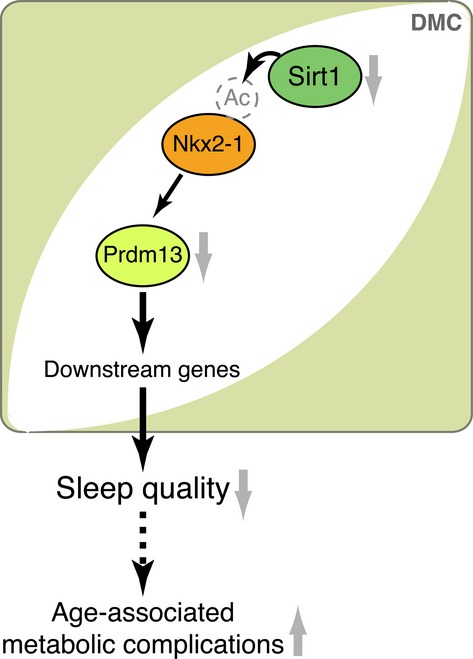
Model for the compact region of the DMH (DMC) in controlling sleep quality and metabolism during aging. The levels of *Prdm13* decline with age in the DMC, causing altered expression of downstream target genes. This age-associated dysfunction of DMC neurons likely reduces sleep quality and causes metabolic complications such as increased adiposity during the aging process. *Prdm13* transcription is upregulated by Nkx2-1, whose activity is regulated by Sirt1-mediated deacetylation. Therefore, the age-associated decline in *Prdm13* expression might be due to the reduction of Sirt1 activity during the aging process.

It is likely that the Nkx2-1/Prdm13 pathway in the DMH has a significant contribution to the Sirt1-mediated control of aging and longevity (Fig.6). At the molecular level, Nkx2-1 is deacetylated by Sirt1 in the hypothalamus during the dark period when Sirt1 is activated (Satoh *et al*., 2013). In addition, Sirt1 cooperates with Nkx2-1 to upregulate *Ox2r* transcription (Satoh *et al*., 2013). Whether Sirt1 is also involved in the regulation of *Prdm13* transcription and whether age-associated reduction of *Prdm13* expression in the hypothalamus is due to a decline in Sirt1 activity still need to be investigated. It will also be important to examine potential roles of downstream targets of Prdm13, Nkx2-1, and Sirt1 in the DMH in the pathogenesis of age-associated sleep abnormality and metabolic complications. We demonstrated that BRASTO mice display delays in age-associated physiological declines and lifespan extension (Satoh *et al*., 2013). Maintaining quality of sleep is one of the striking phenotypes in aged BRASTO mice. In contrast, DMH/LH-specific *Nkx2-1*- or *Sirt1-*knockdown mice (Satoh *et al*., 2013) and DMH-specific *Prdm13*-knockdown mice display lower NREM delta power compared to controls. In addition, Sirt1 and Nkx2-1 are colocalized in the DMC, and *Prdm13* is also localized in the same region. These results strongly imply that Prdm13, Nkx2-1, and Sirt1 together in the DMC play an important role in the regulation of sleep quality during the aging process. On the other hand, the role of DMH neurons expressing Nkx2-1 and/or Prdm13 in the regulation of body weight and adiposity is still unclear. Although DMH-specific *Prdm13*-knockdown mice display progressive weight gain, DMH/LH-specific *Nkx2-1*-knockdown mice do not display such phenotypes (unpublished finding). Much longer observation time or excessive energy intake might be necessary to produce definitive changes in body weight in DMH-specific *Nkx2-1*-knockdown mice. Alternatively, this phenotypic difference between *Nkx2-1*- and *Prdm13*-knockdown mice might be due to the heterogeneity of DMH neurons, particularly DMC neurons, expressing *Nkx2-1*,*Prdm13,* and/or *Sirt1*. In other words, Prdm13 in distinct subpopulations of DMC neurons, each of which are characterized by different neuropeptides or neurotransmitters, might differentially regulate sleep quality and body weight. These distinct neuronal subsets could mediate different functions through different projections but together contribute to the prevention against age-associated physiological decline and the promotion of longevity in mammals.

In conclusion, our microarray-based gene expression profiling for hypothalamic nuclei provided valuable marker genes for the DMH. Investigation of the physiological roles of DMH-enriched genes in sleep homeostasis and metabolism will shed new light on the understanding of aging and longevity control in mammals.

## Experimental procedures

### Animal study

C57BL/6J female mice were purchased from the Jackson Laboratory (Bar Harbor, ME, USA). All mice were maintained under 12-h light/12-h dark conditions and had free access to food and water. For DR, mice were fed 60% of control for 3 months as previously described (Satoh *et al*., 2010). The procedure for stereotactic delivery of lentiviruses was described previously (Satoh *et al*., 2013). Body composition was measured using EchoMRI™ 3-in-1 Body Composition Analyzer (EchoMRI LLC, Houston, TX, USA). BRASTO females in C57BL/6J background were described previously (Satoh *et al*., 2010). All animal procedures were approved by the Washington University Animal Studies Committee and were in accordance with NIH guidelines.

### Sleep analysis

Briefly, isoflurane-anesthetized mice were surgically implanted with screw electrodes in the skull for electroencephalogram (EEG) and platinum wire electrodes in the nuchal muscle for electromyogram (EMG) recording. After a minimum of 2-week recovery, we conducted EEG/EMG recording under each physiological condition. The 10-s epochs of EEG/EMG signals were scored by visual examination as wake, lower amplitude delta (1–4 Hz) and theta (4–8 Hz) frequency with higher and variable amplitude EMG activity; NREM sleep, predominantly higher amplitude delta activity and low amplitude EMG activity; or REM sleep, predominantly low amplitude rhythmic theta activity and very low amplitude EMG activity. The 10-s EEG epochs then were processed by Fourier transformation to calculate the percentage of delta power relative to total power in the 0.1–25 Hz frequency range for each 10-s epoch. A Wilcoxon matched-pairs signed-rank test was used to compare differences of hourly data points through the light and dark periods between groups in sleep analysis.

### Sample collection of hypothalamic nuclei

The Arc, VMH, DMH, LH, PVN, and SCN were dissected from two C57BL/6J females at 3 months of age by laser microdissection using the Leica LMD 6000 system (Leica Microsystems, Buffalo Grove, IL, USA). We confirmed that there was no contamination from surrounding hypothalamic nuclei by conducting quantitative real-time RT-PCR using probes *Agrp*,*Nr5a1*,*hypocretin (Hcrt)*, and *corticotropin-releasing hormone (Crh)* (Fig. S1B). RNA was isolated from each hypothalamic nucleus using the Arcturus PicoPure RNA Isolation Kit (Life Technologies, Grand Island, NY, USA). The detailed procedure was described previously (Satoh *et al*., 2010). For microarray, the quality of total RNA was determined by Agilent 2100 Bioanalyzer (Agilent Technologies, Santa Clara, CA, USA), and two samples for each hypothalamic nucleus were used for microarray analysis. For quantitative real-time RT-PCR, RNA concentration was determined by NanoDrop, and cDNA was synthesized using the Applied Biosystems High-Capacity cDNA Reverse Transcription Kit (Life Technologies, Grand Island, NY, USA).

### Agilent one color microarray and expression analysis

Total RNA was amplified by the WTA2 kit (Sigma-Aldrich, St. Louis, MO, USA), according to the manufacturer's protocol. cDNAs were chemically labeled with Kreatech ULS RNA Labeling Kit (Kreatech Diagnostics, Durham, NC, USA). Labeled cDNA was purified with Qiagen PCR purification columns, and cDNAs were quantitated on a NanoDrop spectrophotometer. cDNA samples were hybridized to Agilent Mouse_v1 8x60K microarrays (Design ID-028005, Agilent Technologies, Santa Clara, CA, USA). Slides were scanned on an Agilent C-class Microarray scanner to detect Cy5 fluorescence, according to the manufacturer's specifications. Gridding and analysis of images was performed using Feature Extraction software (v11.5.1.1, Agilent Technologies, Santa Clara, CA, USA). To identify differentially expressed genes in each hypothalamic nucleus, ratios of signal intensities to each hypothalamic nucleus were calculated, and genes whose expression levels were greater than or equal to 10-fold in each hypothalamic nucleus compared to the others were selected. The heat map for each log2-transformed gene expression level was generated using Partek Genomics Suite (Partek Inc., St. Louis, MO, USA). The microarray data were deposited into the NCBI GEO database (GEO Accession Number GSE58238).

### Quantitative real-time RT-PCR

Total RNA was extracted from brain regions using the Ambion PureLink RNA Mini Kit (Life Technologies, Grand Island, NY, USA). Hypothalamic nuclei were collected by laser microdissection as described above. RNA was reverse-transcribed into cDNA as described above, and quantitative real-time RT-PCR was conducted as described in detail previously (Satoh *et al*., 2013). Relative expression levels of the genes of interest were normalized to *Gapdh* levels.

### Luciferase assay

HEK293 cells were transfected with a firefly luciferase reporter driven by 360-bp *Prdm13* promoter, pCMV-Nkx2-1 (a gift from Dr. Shioko Kimura, National Cancer Institute, Bethesda, MD, USA) or control vector, and a *Renilla* luciferase expression vector. Luciferase assays were conducted as described previously (Satoh *et al*., 2010). Firefly luciferase activity levels were normalized to *Renilla* luciferase activity of each sample.

### Primary hypothalamic neuronal isolation and *Nkx2-1* knockdown

Hypothalami were dissected from E15.5 C57BL/6J mice, and hypothalamic neurons were isolated as described previously (Satoh *et al*., 2013). Hypothalamic neurons were plated at 2.5 × 10^5^ cells/well in 6-well plates coated with 100 μg ml^-1^ poly-L-lysine, and cells were maintained in Neurobasal media containing 10% FBS, 1 ×  B27, 2 mm L-glutamine, and penicillin/streptomycin. Cultures were treated with 10 μm Ara-C (Sigma-Aldrich, St. Louis, MO, USA) after 2 days *in vitro* (DIV). Media was replaced to Dulbecco's modified Eagle's media containing 50% F12, 5 mm HEPES, 1x B27, 1x N2, 0.5% L-glutamax, 0.5 mM sodium pyruvate, and penicillin/streptomycin after 4 DIV. On the next day, *Nkx2-1* shRNA lentivirus was added, and cells were harvested after 7 DIV. RNA was isolated as described previously (Satoh *et al*., 2013).

### Statistical analysis

Statistical analyses were carried out using unpaired or paired Student's *t*-tests for two groups and one-way ANOVA with the Tukey–Kramer *post hoc* test for more than two groups. The rhythmicity of gene expression was assessed by the JTK-Cycle (Hughes *et al*., 2010), which is available as a computationally efficient R script (http://openwetware.org/wiki/HughesLab:JTK_Cycle). In all analyses, we set the α level at 0.05. We used Microsoft Excel 2008 and SPSS 11.0 to conduct statistical analyses.

## Supporting Information

**Table S1** Genes selectively expressed in the Arc, VMH, DMH, and LH

**Data S1** Supplementary Figure Legends and Experimental Procedures.

**Fig. S1** Laser-microdissection of distinct hypothalamic nuclei.

**Fig. S2** Nkx2-1 is colocalized with NeuN in the DMH.
