# Supplementary material for: Deficiency of Prdm13, a dorsomedial hypothalamus-enriched gene, mimics age-associated changes in sleep quality and adiposity
Source: Aging Cell. 2014 Dec 25;14(2):209–18. doi: 10.1111/acel.12299 (PMC4364833; doi:10.1111/acel.12299)
Supplement: Supplementary file 2 [file acel0014-0209-sd2.docx]

**Supporting Information**

**Figure legends**

**Fig. S1.** Laser-microdissection of distinct hypothalamic nuclei. (A) Nissl staining of hypothalamic sections at Bregma -1.46 mm (left) and Bregma -1.82 mm (right) before and after laser microdissection (top and middle, respectively). Nissl substance in the cytoplasm of neurons is indicated as purple staining. The outlines in the bottom panels indicate the Arc, VMH, LH, and DMH subdivisions (DMD, DMC, and DMV). (B) RNA expression levels of *Agrp*, *Nr5a1, Hcrt*, and *Crh* mark the Arc, VMH, LH, and PVN, respectively, confirming there is no contamination from surrounding nuclei. Results are shown as mean ± S.E. (n= 2-3 mice for each group).

**Fig. S2.**  Nkx2-1 is colocalized with NeuN in the DMH. (A-C) Immunofluorescence of Nkx2-1 (A), NeuN (B) and merged image (C) in the DMH. (D) Higher magnification shows significant overlap of Nkx2-1 with NeuN.

**Experimental Procedures**

**Immunofluorescence microscopy**

Immunofluorescent staining was performed as previously described (Satoh et al., 2010).

Briefly, samples were stained first with anti-Nkx2-1 (TTF-1) (1:500, Santa Cruz Biotechnology, Inc., Dallas, TX, USA) and Dylight 594-conjugated goat anti-rabbit IgG (1:200, Jackson ImmunoResearch Laboratories, Inc., West Grove, PA, USA), and then stained with anti-NeuN (1:500, Millipore Corporation, Temecula, CA, USA) and Dylight 488-conjugated goat anti-mouse IgG (1:500, Jackson ImmunoResearch Laboratories, Inc., West Grove, PA, USA). Images of Nkx2-1 staining alone were taken on a Zeiss Axio Imager.Z1 microscope (Carl Zeiss, Jena, Germany). Images of Nkx2-1/NeuN staining were acquired on a Nikon Eclipse C1 Confocal microscope (Nikon Instruments Inc., Melville, NJ, USA) using 20X or 40X oil objectives. Images shown were maximal Z-projections of confocal stacks.
