# Supplementary figures and images for: Deficiency of Prdm13, a dorsomedial hypothalamus-enriched gene, mimics age-associated changes in sleep quality and adiposity
Source: Aging Cell. 2014 Dec 25;14(2):209–18. doi: 10.1111/acel.12299 (PMC4364833; doi:10.1111/acel.12299)

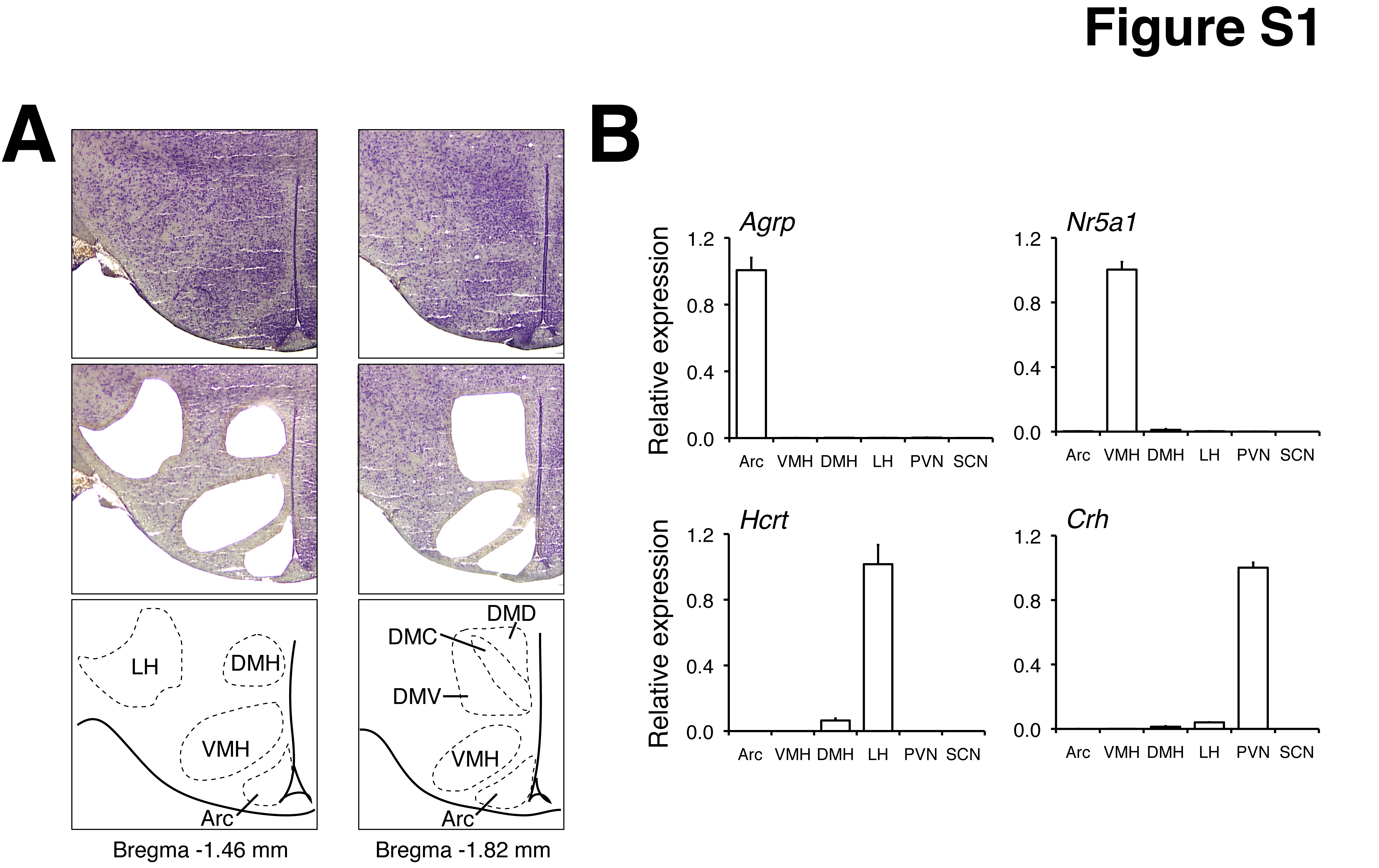

Supplement: Supplementary file 3 [file acel0014-0209-sd3.tif]

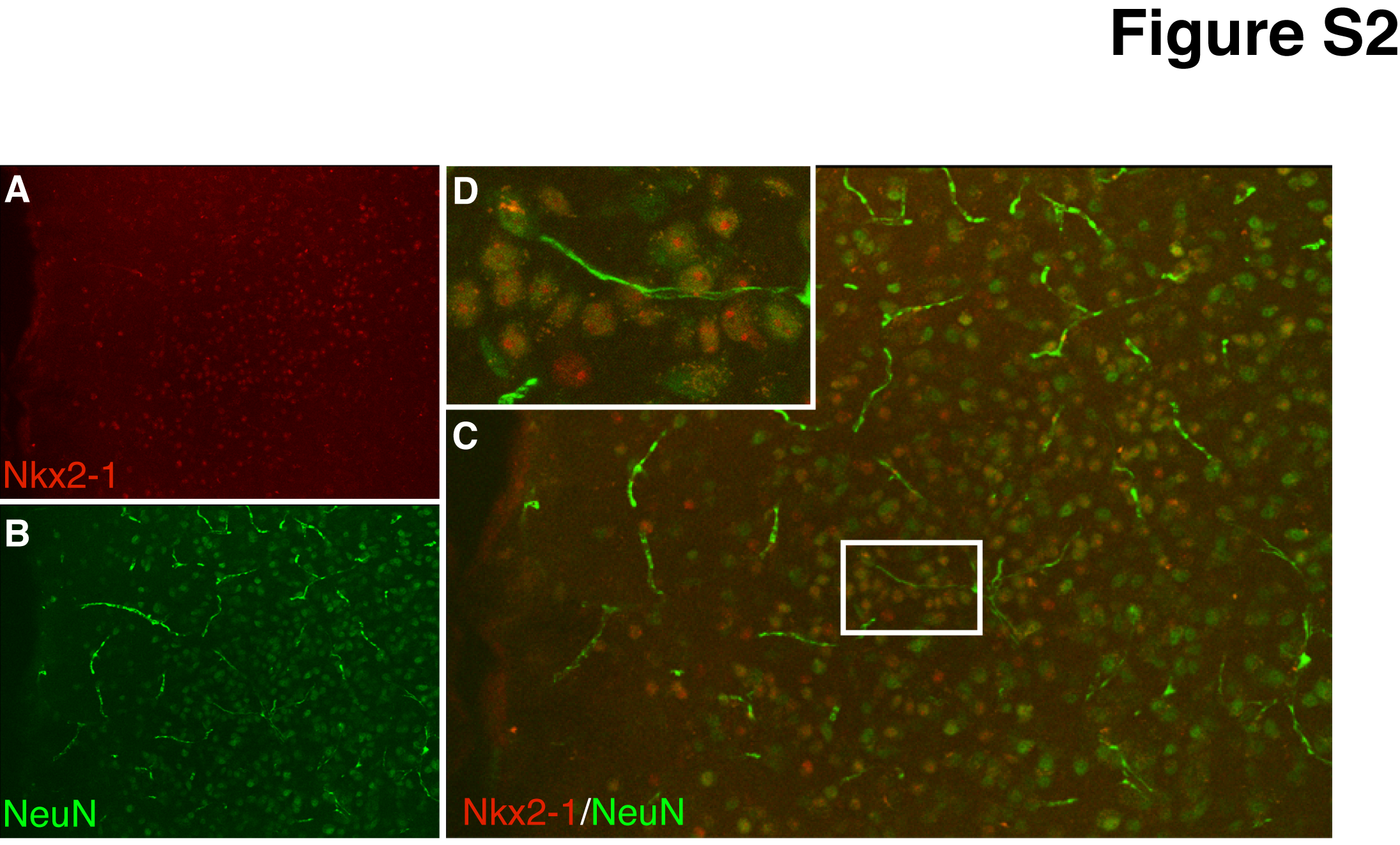

Supplement: Supplementary file 4 [file acel0014-0209-sd4.tif]
